# Effects of five cannabis oils with different CBD: THC ratios and terpenes on hypertension, dyslipidemia, hepatic steatosis, oxidative stress, and CB1 receptor in an experimental model

**DOI:** 10.1186/s42238-025-00286-8

**Published:** 2025-07-14

**Authors:** Valentina Degrave, Michelle Berenice Vega Joubert, Camila Filippa, Paola Ingaramo, Lucía Torregiani, Yamile Soledad Caro, María Mercedes De Zan, María Eugenia D’Alessandro, María Eugenia Oliva

**Affiliations:** 1 Laboratorio de Estudio de Enfermedades Metabólicas relacionadas con la Nutrición, Facultad de Bioquímica y Ciencias Biológicas, Universidad Nacional del Litoral, Santa Fe, Argentina; 2 Consejo Nacional de Investigaciones Científicas y Técnicas (CONICET), Santa Fe, Argentina; 3 Instituto de Salud y Ambiente del Litoral (ISAL), Facultad de Bioquímica y Ciencias Biológicas, Consejo Nacional de Investigaciones Científicas y Técnicas (CONICET), Santa Fe, Argentina; 4 Laboratorio de Control de Calidad de Medicamentos, Facultad de Bioquímica y Ciencias Biológicas, Universidad Nacional del Litoral., Santa Fe, Argentina; 5 Facultad de Bioquímica y Ciencias Biológicas, Universidad Nacional del Litoral. Ciudad Universitaria, Santa Fe, cc242 (3000) Argentina

**Keywords:** Cannabis oil, Cannabinoid, Terpenes, Hepatic steatosis, Oxidative stress

## Abstract

**Background:**

Non-alcoholic fatty liver disease (NAFLD) is a common liver disorder caused by oxidative stress and dysregulation of lipid metabolism. The endocannabinoid system (ECS), particularly the type 1 cannabinoid (CB1) receptor, plays a crucial role in NAFLD progression. Cannabinoids, such as cannabidiol (CBD) and tetrahydrocannabinol (THC), along with terpenes, such as beta-myrcene and d-limonene, have shown potential therapeutic effects on liver health, particularly in reducing oxidative stress and modulating lipid metabolism. This study aimed to analyse the effects of five cannabis oils (COs), each with different CBD:THC ratios and terpenes content, on hypertension, dyslipidemia, hepatic steatosis, oxidative stress, and CB1 receptor expression in an experimental model of NAFLD induced by a sucrose-rich diet (SRD) in Wistar rats for 3 weeks.

**Methods:**

Male Wistar rats were fed either a: (1) reference diet (RD; standard commercial laboratory diet) or a: (2) sucrose-rich diet (SRD) for 3 weeks. 3 to 7 SRD + CO as following: (3) SRD + THC; (4) SRD + CBD; (5) SRD + CBD:THC 1:1; (6) SRD + CBD:THC 2:1; and (7) SRD + CBD:THC 3:1. The COs were administered orally at a dose of 1.5 mg total cannabinoids/kg body weight daily. The cannabinoid and terpenes content of all COs used in the study was determined. The terpenes found in COs were beta-myrcene, d-limonene, terpinolene, linalool, beta-caryophyllene, alpha-humulene, (-)-guaiol, (-)-alpha-bisabolol. During the experimental period, body weight, food intake and blood pressure were measured. Serum glucose, triglyceride, total cholesterol, uric acid, alanine aminotransferase (ALT), aspartate aminotransferase (AST), and alkaline phosphatase (AP) levels were evaluated. Liver tissue histology, NAFLD activity score (NAS), triglyceride and cholesterol content, lipogenic enzyme activities, enzyme related to mitochondrial fatty acid oxidation, reactive oxygen species (ROS), thiobarbituric acid reactive substance (TBARS), and antioxidant enzyme activities were also evaluated. The CB1 receptor expression was also determined.

**Results:**

The results showed that SRD-fed rats developed hypertension, dyslipidemia, liver damage, hepatic steatosis, lipid peroxidation, and oxidative stress. This was accompanied by upregulation of liver CB1 receptor expression. CBD-rich CO, CBD:THC 1:1 ratio CO; CBD:THC 2:1 ratio CO and CBD:THC 3:1 ratio CO showed antihypertensive properties. THC-rich CO, CBD:THC 1:1 ratio CO; CBD:THC 2:1 ratio CO showed the greatest beneficial effects against hepatic steatosis and liver damage. All COs exhibited antioxidant effects in liver tissue. This was associated with normal liver CB1 receptor expression.

**Conclusions:**

This study demonstrated that COs, particularly THC-rich CO, CBD:THC ratio 1:1 CO, CBD:THC ratio 2:1 CO and terpenes, can effectively reduce dyslipidemia, liver damage and hepatic steatosis in SRD-induced NAFLD. COs with a higher proportion of CBD in their composition showed antihypertensive properties. All the COs exhibited antioxidant properties. These findings suggest that COs, especially those with CBD:THC ratios of 1:1 and 2:1 and terpenes, may represent a promising therapeutic approach for managing NAFLD and preventing its progression to more severe liver disease.

**Supplementary Information:**

The online version contains supplementary material available at 10.1186/s42238-025-00286-8.

## Introduction

Non-alcoholic fatty liver disease (NAFLD) is one of the most common liver disorders globally and is characterized by excessive fat accumulation in the liver unrelated to alcohol consumption. NAFLD encompasses a spectrum ranging from benign hepatic steatosis to non-alcoholic steatohepatitis (NASH) and cirrhosis, which significantly increases the risk of liver failure and cardiovascular disease. Oxidative stress, inflammation, and dysregulation of lipid metabolism are central to the progression of NAFLD, making it a critical area of focus in the search for effective therapeutic interventions (Allameh et al. 2023; Chen et al. 2020; Friedman et al. 2018; Buzzetti et al. 2016).

Oxidative stress is a key contributor to liver damage in NAFLD and is driven by an imbalance between reactive oxygen species (ROS) production and antioxidant defences in the liver. This process exacerbates lipid peroxidation, mitochondrial dysfunction, and inflammatory responses, all of which drive to hepatic injury. Recent studies have highlighted the role of the endocannabinoid system (ECS), particularly the CB1 receptor, in promoting hepatic steatosis, inflammation, and oxidative stress, suggesting that CB1 receptor modulation could be a valuable strategy in managing NAFLD (Aloisio Caruso et al. 2025; Kurtov et al. 2024; Charytoniuk et al. 2022; Mboumba Bouassa et al. 2022; Berk et al. 2021; Jorgačević et al. 2021; Chang et al. 2018; Mallat et al. 2013; Silvestri and Di Marzo 2013; Osei-Hyiaman et al. 2005).

*Cannabis sativa* and its derivatives, particularly the cannabinoids cannabidiol (CBD) and delta-9-tetrahydrocannabinol (THC), have attracted increasing interest for their potential therapeutic effects in various metabolic and liver-related diseases. CBD and THC have gained attention because of their potential to modulate liver metabolism and reduce oxidative stress. However, the therapeutic effects of these cannabinoids can be enhanced in the presence of terpenes, which are naturally aromatic compounds in cannabis. Terpenes such as beta-myrcene, d-limonene, terpinolene, beta-caryophyllene and alpha-humulene have demonstrated anti-inflammatory, antioxidant, and hepatoprotective properties, which may complement the actions of cannabinoids (Chen and Kim 2024; Pagano et al. 2023; Ghasemi-Gojani et al. 2022; Nuutinen 2018; Russo 2011).

In this study, we investigated the effects of five COs, each with different CBD: THC ratios and significant terpene content, on hypertension, dyslipidemia, hepatic steatosis, oxidative stress, and CB1 receptor expression in an experimental model of NAFLD induced by a sucrose-rich diet (SRD) in Wistar rats for 3 weeks. By examining the combined impact of cannabinoids and terpenes, this study sought to provide new insights into the therapeutic potential of cannabis-derived compounds in the treatment of NAFLD.

## Materials and methods

### Cannabis oils Preparation

COs were obtained from dried inflorescences of the *Cannabis sativa* Aromito and Alfajor Zkittlez varieties grown at NISOR SRL, Santa Fe, Argentina (RNCyFS 11526 AFH). Briefly, the dried inflorescences were placed at -4 °C for 24 h. After that, an alcoholic extraction (10 ml ethanol 96º per gram of inflorescence) was carried out, and subsequently, the ethanol was evaporated at low temperature with a rotavapor (FIGMAY RV5) to obtain the resin. The resin was transferred to a magnetic stirrer and the cannabinoids were decarboxylated at 110–120 °C for 10 min. Subsequently, the resin was diluted in corn oil to achieve an estimated cannabinoid concentration of 2% (w/v). The two resins were utilized to prepare 5 COs, designed as THC-rich CO, CBD-rich CO, CBD:THC 1:1 ratio CO, CBD:THC 2:1 ratio CO, and CBD:THC 3:1 ratio CO. The cannabinoids and the terpenes were quantified using chromatographic methods, and appropriate dilutions were carried out in corn oil to obtain the working oils, as shown in Tables 1 and 2.

**Table 1:** Quali-quantitative cannabinoid profiles in working oils

Cannabinoid	Cannabinoids concentration (mg/mL) in COs				
	THC-rich	CBD-rich	CBD:THC 1:1 ratio	CBD:THC 2:1 ratio	CBD:THC 3:1 ratio
Cannabidivarinic acid (CBDVA)	ND	ND	ND	ND	ND
Cannabidivarin (CBDV)	ND	ND	0.01	0.01	0.02
Cannabidiolic acid (CBDA)	ND	ND	0.01	0.01	0.01
Cannabigerolic acid (CBGA)	ND	ND	ND	ND	ND
Cannabigerol (CBG)	0.10	0.10	0.05	0.05	0.04
Cannabidiol (CBD)	0.35	2.70	0.69	0.92	1.07
Tetrahydrocannabivarin (THCV)	ND	ND	ND	ND	ND
Tetrahydrocannabivarinic acid (THCVA)	ND	ND	ND	ND	ND
Cannabinol (CBN)	0.04	ND	0.01	0.01	0.01
Δ-9-Tetrahydrocannabinol (Δ-9-THC)	2.63	0.14	0.71	0.48	0.31
Δ-8-Tetrahydrocannabinol (Δ-8-THC)	ND	ND	ND	ND	ND
Cannabichromene (CBC)	0.05	0.11	0.04	0.05	0.06
Tetrahydrocannabinolic acid (THCA)	ND	ND	ND	ND	ND
**Total CBD**	0.35	2.70	0.70	0.93	1.08
**Total THC**	2.63	0.14	0.71	0.48	0.31
**Total Cannabinoids**	3.17	3.06	1.52	1.53	1.51
**Ratio CBD:THC**	1.0:7.5	1.0:0.05	1.0:1.0	2.0:1.0	3.5:1.0

**ND**: not detected

Total CBD = (CBD + CBDA x 0.877). Total THC = (THC + THCA x 0.877)

**Table 2:** Quali-quantitative terpene profiles in working oils

Terpenes	Terpenes concentration (mg/L) in COs				
	THC-rich	CBD-rich	CBD:THC 1:1 ratio	CBD:THC 2:1 ratio	CBD:THC 3:1 ratio
alpha-pinene	ND	ND	ND	ND	ND
camphene	ND	ND	ND	ND	ND
(-)-beta-pinene	ND	ND	ND	ND	ND
beta-myrcene	0.14	1.57	1.02	1.08	1.11
delta-3-carene	ND	ND	ND	ND	ND
alpha-terpinene	ND	ND	ND	ND	ND
p-cymene	ND	ND	ND	ND	ND
d-limonene	0.24	0.50	0.50	0.42	0.50
cis-b-ocimene	ND	ND	ND	ND	ND
trans-b-ocimene	ND	ND	ND	ND	ND
gamma-terpinene	ND	ND	ND	ND	ND
terpinolene	0.96	ND	0.79	0.48	0.36
linalool	1.61	1.54	2.29	1.70	1.52
(-)-isopulegol	ND	ND	ND	ND	ND
geraniol	ND	ND	ND	ND	ND
beta-caryophyllene	13.81	10.56	15.89	11.63	11.13
alpha-humulene	4.44	3.96	5.71	4.17	5.18
cis-nerolidol	ND	0.71	ND	ND	ND
trans-nerolidol	ND	ND	ND	ND	ND
(-)-guaiol	3.68	7.40	7.00	6.17	6.16
(-)-alpha-bisabolol	2.46	13.17	10.74	9.93	10.73
**Total terpenes**	**27.34**	**39.41**	**43.95**	**35.59**	**36.69**

**ND**: not detected

### Cannabis oils characterization: cannabinoids and terpenes

#### Qualitative and quantitative profiles of cannabinoids

**Fig. 1:**
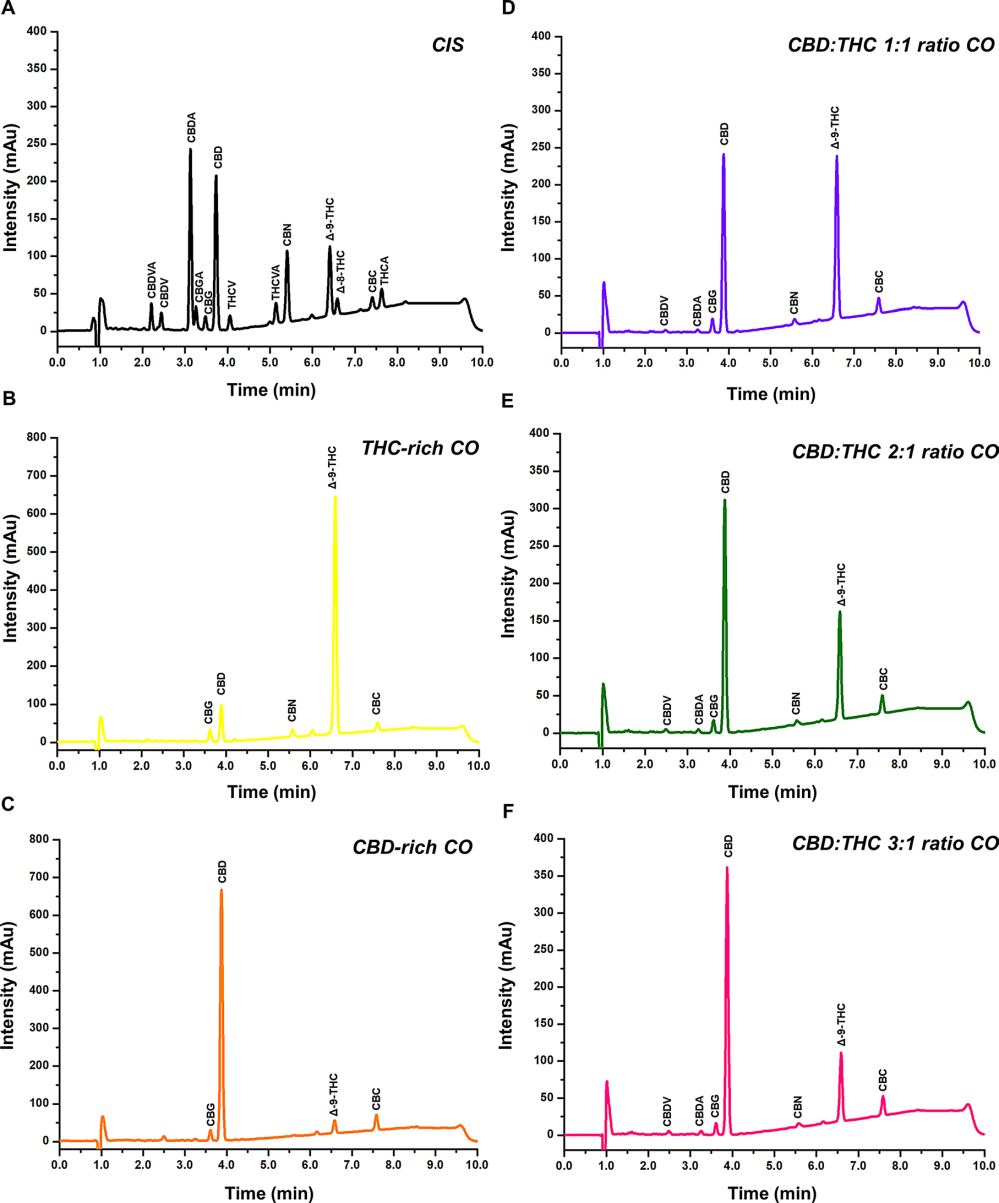
Cannabinoids oils profiles. (**A**) Cannabinoid identification solution (CIS): CBDVA (cannabidivarinic acid), CBDV (Cannabidivarin), CBDA (Cannabidiolic acid), CBGA (Cannabigerolic acid), CBG (Cannabigerol), CBD (Cannabidiol), THCV (Tetrahydrocannabivarin), THCVA (Tetrahydrocannabivarinic acid), CBN (Cannabinol), Δ-9-THC (Delta-9-Tetrahydrocannabinol), Δ-8-THC (Delta-8-Tetrahydrocannabinol), THCA (Tetrahydrocannabinolic acid), and CBC (Cannabichromene). (**B**-**F**) Chromatographic profiles of CBD-rich CO, CBD:THC 1:1 ratio CO; CBD:THC 2:1 ratio CO and CBD:THC 3:1 ratio CO

The cannabinoids profiles of the different oils were determined by high-performance liquid chromatography with diode array detection (HPLC-DAD) using an Agilent 1260 series system (Analytical Tech, Germany) equipped with a quaternary pump, a membrane degasser, a thermostated column compartment, and an autosampler. Certified reference materials for cannabinoids were obtained from the United States Pharmacopeia and the Restek Corporation. The analytical method was adapted from Sarma et al. (2020) and fully validated according to the recommendations of the ICH Q2R (2) guidelines (2022). Briefly, cannabinoids were extracted with ethanol from an aliquot of 50 µL of CO using a combination of sonication, vortex agitation, and centrifugation. Chromatographic separation was achieved using an Infinity Lab Poroshell 120 EC-C18 column (4.6 × 150 mm, 2.7 μm particle size) and a binary gradient for the mobile phase (solvent A: water with 0.1% formic acid, and solvent B: acetonitrile with 0.1% formic acid). Initial conditions were 74% B for 2.5 min, increased to 85% B over the next 4 min, held at 85% B for 1.5 min, decreased to 74% B in 0.1 min, and held at 74% B for 2.0 min to re-equilibrate of the system. The flow rate was 1.5 mL/min and the column compartment temperature was set at 40 °C. The injection volume was 5.0 µL, and the DAD was set to record chromatograms at 220 nm. Figure 1 shows the COs cannabinoids profiles.

#### Qualitative and quantitative profiles of terpenes

**Fig. 2:**
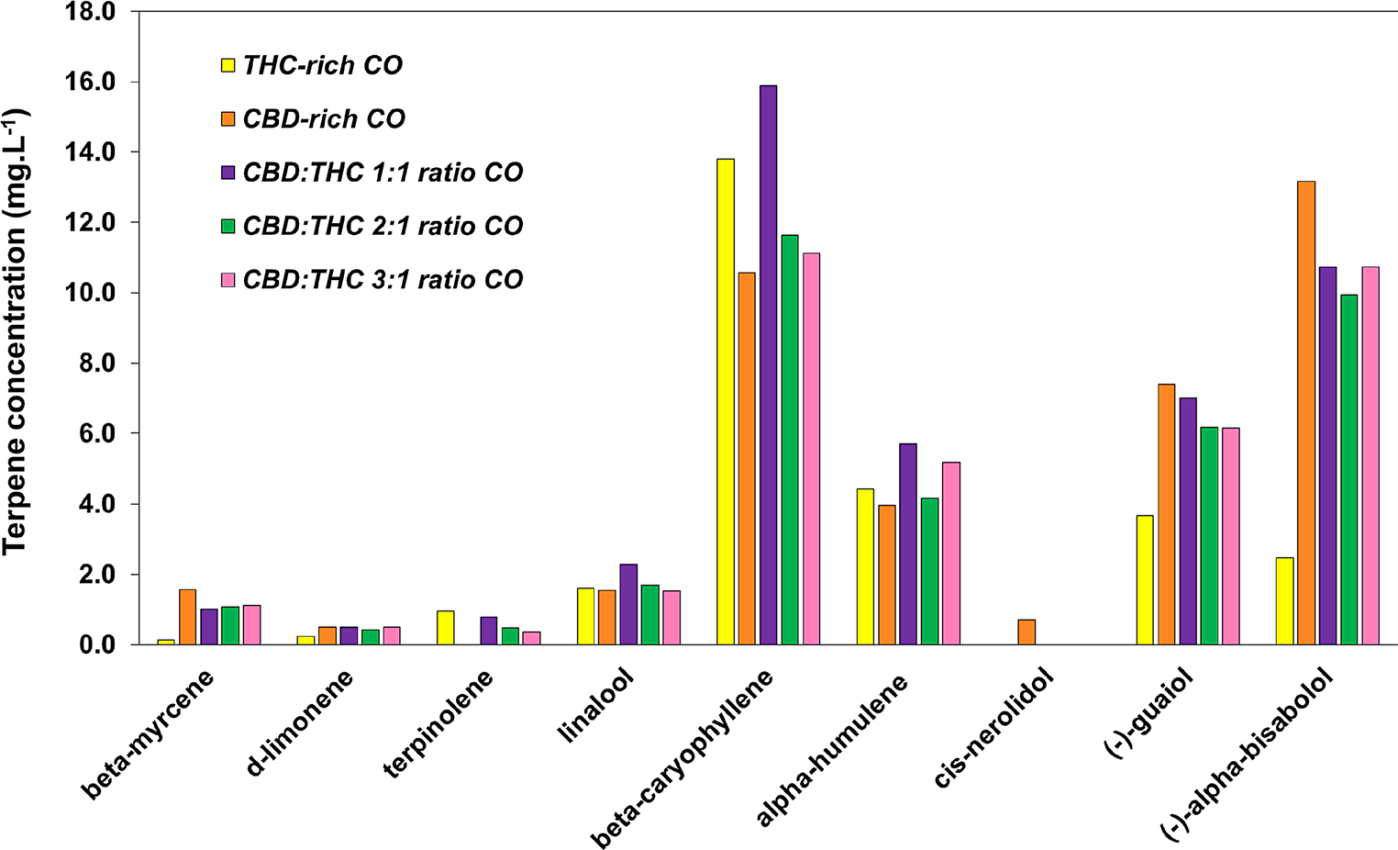
Terpenes oils profiles. Terpenes contents of CBD-rich CO, CBD:THC 1:1 ratio CO; CBD:THC 2:1 ratio CO and CBD:THC 3:1 ratio CO

The terpenes profiles were determined using the same ethanolic extract as used for the cannabinoids, employing a gas chromatography instrument coupled to a mass spectrometry detector (GCMS-QP2020 NX, Shimadzu, Japan). A terpenes mixture solution, certified reference material (Restek) including *Cannabis sativa* characteristics monoterpenes and sesquiterpenes (21 terpenes in total) was employed for method calibration. Separation was achieved as described by Chua et al. (2018) using a capillary column (30 m x 0.25 mm ID) with a 0.25 μm film thickness, composed of 5% diphenyl and 95% dimethyl polysiloxane (Restek) with helium as the carrier gas in a linear velocity mode. Aliquots of 0.5 µl of sample or standard solutions were injected into the chromatograph with the autosampler set at 250 °C and a 10:1 split ratio. The oven temperature was initially held at 55 °C for 0–2 min, increased to 125 °C at a rate of 8 °C/min, and then increased to 280 °C at 30 °C/min, with a final hold time of 3 min. The ion source and interface temperatures were set at 220 and 260 °C, respectively. Data were acquired in full scan mode (50–500 m/z mass range) and selected ion monitoring (SIM) mode for quantitative purposes. Figure 2 shows the COs terpenes profiles. The terpenes found in COs were beta-myrcene, d-limonene, terpinolene, linalool, beta-caryophyllene, alpha-humulene, (-)-guaiol, (-)-alpha-bisabolol.

### Animals and diets

Male Wistar rats (*n* = 42) purchased from the Veterinary Sciences Institute of Litoral (Instituto de Ciencias Veterinarias del Litoral, ICIVET-Litoral) -Facultad de Veterinaria, Universidad Nacional del Litoral (Esperanza, Santa Fe, Argentina) were maintained with unrestricted access to water and food under controlled temperature (22 ± 1 °C), humidity and air flow conditions, with a fixed 12-h light/dark cycle (light on 07.00 a.m to 7.00 p.m). Adequate measures were taken to minimize the pain or discomfort of the rats and we used the smallest number of animals possible. This study was performed in strict accordance with the NIH guidelines for the care and use of laboratory animals and was approved by the Institutional Ethics Committee of the Faculty of Biochemistry and Biological Sciences (UNL, Santa Fe, Argentina - Acta 2/22).

The animals were initially fed a standard powdered rodent commercial diet (GEPSA FEED, Buenos Aires, Argentina). When the rat’s weight was 180–190 g, were fed for 3 weeks with either: (1) a reference diet (RD; standard commercial laboratory diet) or (2) a sucrose-rich diet (SRD). In addition, five experimental groups received the SRD with different COs: (3) Rats fed a SRD plus oral administered THC-rich CO (SRD + THC), *n* = 6; (4) Rats fed a SRD plus oral administered CBD-rich CO (SRD + CBD), *n* = 6; (5) Rats fed a SRD plus oral administered CBD:THC 1:1 ratio CO (SRD + CBD:THC 1:1), *n* = 6; (6) Rats fed a SRD plus oral administered CBD:THC 2:1 ratio CO (SRD + CBD:THC 2:1), *n* = 6 and (7) Rats fed a SRD plus oral administered CBD:THC 3:1 ratio CO (SRD + CBD:THC 3:1), *n* = 6. COs or vehicle (corn oil for RD and SRD groups) was administered for 3 weeks at a dose of 1.5 mg total cannabinoids/kg body weight. COs or vehicle were administered noninvasively using an oral syringe. This method is widely employed in preclinical studies due to its speed, ability to ensure accurate dosing, capacity to minimize stress, and potential to enhance animal welfare (Chesler et al. 2022; Tillmann and Wegener 2018; Turner et al. 2011). This dose was slightly higher than the 1.0 mg/kg dose used in our previous studies (Degrave et al. 2023a, Degrave et al. 2023b), where promising results were observed using a single CO formulation. To further explore these findings, the current study evaluates the effects of five distinct CO formulations, each characterized by different CBDTHC ratios and terpenes content.

The diet compositions are detailed in a study by Degrave et al. (2023a). Individual body weight was recorded daily. Food intake of the animals in each group were assessed twice a week throughout the experimental period. At the end, the food was removed at 07.00 a.m. and experiments were performed between 07.00 and 09.00 a.m. The animals were anesthetized with intraperitoneal sodium pentobarbital (60 mg/kg body weight). Blood samples were collected from the inferior vena cava, rapidly centrifuged and serum was either immediately assayed or stored at − 20 °C until used. The liver of each rat was totally removed, weighed, and sectioned for different subsequent assays. Liver samples were fixed in 10% (v/v) buffered formalin for 24 h at room temperature and embedded in paraffin for histology analysis or frozen and stored at the temperature of liquid N_2_. Finally, the animals were euthanized by removal of vital organ (heart).

### Determination of blood pressure

Blood pressure was measured in the three dietary groups in conscious animals during the experimental period using a CODA™ Monitor of tail-cuff non-invasive blood pressure system (Kent Scientific Corporation, Torrington, CT, USA) as previously described (Fortino et al. 2017).

### Analytical methods

Serum triglyceride, cholesterol, uric acid and glucose levels were measured by spectrophotometric methods using commercial enzymatic kits according to the manufacturer’s protocols (Wiener Lab., Argentina). The activities of serum aspartate aminotransferase (AST), alanine aminotransferase (ALT) and alkaline phosphatase (AP) enzymes were measured by spectrophotometric methods using commercial enzymatic kits according to the manufacturer’s protocols (Wiener Lab., Argentina).

Homogenized liver tissue was used to determine triglyceride and total cholesterol content. Lipid extraction was performed using a chloroform-methanol (2:1) mixture. Aliquots were evaporated and total cholesterol and triglycerides were determined using the enzymatic methods mentioned previously. Lipid peroxidation was assessed by measuring thiobarbituric acid reactive substances (TBARS), and intracellular reactive oxygen species (ROS) in liver tissue were quantified, both as described by Degrave et al. (2023a).

### Liver histology

A semi-automatic rotary microtome (Leica ^®^M2255) was used to obtain cross-sections paraffin-embedded (5 μm thickness) that were stained with hematoxylin-eosin (H&E) to provide an overall view of the tissue. The images of the stained sections were taken under a bright field microscope (Olympus BH2, Tokyo, Japan) with 20X objective. Analysis of images was performed using the software Image Pro-Plus 5.0.2.9 system (Media Cybernetics, Silver Spring, MD, USA). The degree of liver steatosis was assessed semiquantitatively as the percentage of hepatocytes involved. The NAFLD activity score (NAS) was performed as we described previously in Vega Joubert et al. (2022).

### Enzymatic activity assays

Liver Acetyl CoA carboxylase (ACC), Fatty acid synthase (FAS), glucose-6-phosphate dehydrogenase (G-6-PDH) and malic enzyme (ME) activities were assayed as previously described (Degrave et al. 2023a). Liver activity of carnitine palmitoyltransferase-1 (CPT-1) was determined as previously described (Degrave et al. 2023a).

### Antioxidant defense system

Liver glutathione (GSH), a non-enzymatic antioxidant of the hepatic antioxidant defense system, and the activities of different antioxidant enzymes, namely catalase (CAT), glutathione reductase (GR), and glutathione peroxidase (GPx), were determined as previously described (Degrave et al. 2023a).

### Western blot analysis

Frozen liver samples were homogenized in RIPA buffer supplemented with protease inhibitors, left on ice for 10 min, and the supernatant was collected by centrifugation at 25.000 × g for 10 min, as described by Li et al. (2021). Proteins were separated by SDS-PAGE in a 10% gel and electrotransferred onto PVDF membranes. The membranes were probed with mouse primary monoclonal antibodies against CB1 receptor (mouse monoclonal antibody; sc-293419; Santa Cruz Biotecnology) and then incubated with goat anti-mouse IgG conjugated to horseradish peroxidase antibody (mIgG-Fc-BP-HRP; sc-525409, Santa Cruz Biotechnology). Specific signals were visualized using a chemiluminescent detection system (Bio-Lumina, Productos Bio-Logicos, Argentina) according to the manufacturer’s instructions. The intensity of the bands was quantified using optical densitometry (Scion Image Release Beta 4.0.2, NIH, USA). After densitometry of immunoblots, values of the RD group were normalized to 100%, and both SRD and SRD + Ca were expressed relative to this. The protein levels were normalized to those of β-actin.

### Statistical analysis

Results were expressed as mean ± standard error of the mean (SEM). Statistical comparisons were made transversely between different dietary groups using GraphPad Prism 8.0.1. Data were tested for variance using Levene’s test and normality by Shapiro–Wilk’s test. The statistical difference between groups (RD, SRD, SRD + THC, SRD + CBD, SRD + CBD:THC 1:1; SRD + CBD:THC 2:1 and SRD + CBD:THC 3:1) was determined by one-way ANOVA followed by post-hoc Newman-Keuls’ test. P values lower than 0.05 were considered to be statistically significant.

## Results

### Body weight, food intake and systolic and diastolic blood pressure

Table 3 shows that there was no significant difference (*P* < 0.05) in the initial, final body weights and weight gain of the animals in the different experimental groups. Final food intake did not differ between the groups. Final systolic and diastolic blood pressure showed a significantly increase (*P* < 0.05) in SRD-fed rats and SRD + THC group compared with the other groups. CBD-rich CO, CBD:THC 1:1 ratio CO, CBD:THC 2:1 ratio CO and CBD:THC 3:1 ratio CO administration resulted in a significant reduction (*P* < 0.05) in both parameters, compared to the SRD group, reaching reference values.

**Table 3:** Body weight, food intake and systolic and diastolic blood pressure

	RD	SRD	SRD + THC	SRD + CBD	SRD + CBD:THC 1:1	SRD + CBD:THC 2:1	SRD + CBD:THC 3:1
Initial body weight (g)	193.87 ± 1.25	194.58 ± 0.14	195.67 ± 0.80	192.08 ± 0.31	194.83 ± 0.48	187.57 ± 0.72	188.43 ± 1.46
Final body weight (g)	290.87 ± 6.87	294.58 ± 3.68	294.83 ± 2.44	293.33 ± 3.14	295.67 ± 4.18	285.28 ± 2.71	290.57 ± 2.62
Weight gain (g)	97.00 ± 6.04	100.00 ± 3.71	99.17 ± 2.53	101.25 ± 2.71	100.83 ± 4.09	97.71 ± 2.74	102.14 ± 2.03
Food intake (g/day)	17.56 ± 0.38	17.67 ± 0.34	17.87 ± 1.01	17.51 ± 0.72	18.10 ± 0.20	18.04 ± 0.69	18.42 ± 0.45
Diastolic blood pressure (mmHg)	87.10 ± 0.69^b^	93.24 ± 1.18^a^	94.50 ± 0.48^a^	86.48 ± 0.70^b^	87.79 ± 0.59^b^	84.38 ± 0.45^b^	84.18 ± 0.48^b^
Systolic blood pressure (mmHg)	127.30 ± 0.69^b^	136.30 ± 0.91^a^	134.70 ± 0.64^a^	127.6 ± 0.54^b^	126.20 ± 0.49^b^	124.19 ± 0.57^b^	124.38 ± 0.56^b^

Values are expressed as mean ± SEM, *n* = 6. Values in a line that do not share the same superscript letter are significantly different (*P* < 0.05) when one variable at a time was compared by one-way ANOVA followed by Newman-Keuls´ test

Diastolic blood pressure (mmHg): F (DFn, DFd): 3.888 (4, 261), P value: 0.0044; Systolic blood pressure (mmHg): F (DFn, DFd); 3.507 (4, 272), P value: 0.0082

### Serum metabolites

Serum triglyceride, total cholesterol, uric acid, AST, ALT and AP levels were significantly higher in SRD-fed rats compared to RD-fed rats. In the SRD + CBD group serum triglyceride and uric acid decreased only slightly and did not reach reference values. In the SRD + THC, SRD + CBD:THC 1:1, SRD + CBD:THC 2:1 and SRD + CBD:THC 3:1 groups these parameters decreased significantly (*P* < 0.05), reaching reference values. Total cholesterol levels were significantly decreased (*P* < 0.05) in all experimental groups treated with CO. Serum liver damage enzymes, reaching reference values. AST and ALT were significantly increased in SRD, SRD + CBD and SRD + CBD:THC 3:1 groups. THC-rich CO, CBD:THC 1:1 ratio CO and CBD:THC 2:1 ratio CO administration prevented this increase, finding values ​​similar to the RD group. AP enzymes were increased in the SRD group. All COs reduced this increase although without reaching reference values. No changes in serum glucose levels were observed among the dietary groups (Table 4).

**Table 4:** Serum metabolites

	RD	SRD	SRD + THC	SRD + CBD	SRD + CBD:THC 1:1	SRD + CBD:THC 2:1	SRD + CBD:THC 3:1
Glucose (mM)	8.83 ± 0.21	8.86 ± 0.14	9.32 ± 0.2	9.10 ± 0.15	9.53 ± 0.17	9.26 ± 0.29	9.71 ± 0.31
Triglycerides (mM)	1.54 ± 0.06^c^	3.00 ± 0.10^a^	1.62 ± 0.36^c^	2.49 ± 0.28^b^	1.70 ± 0.05^c^	1.65 ± 0.11^c^	1.81 ± 0.09^c^
Total cholesterol (mM)	2.38 ± 0.05^b^	2.86 ± 0.04^a^	2.37 ± 0.08^b^	2.60 ± 0.06^b^	2.58 ± 0.06^b^	2.43 ± 0.02^b^	2.61 ± 0.09^b^
Uric acid (mM)	70.24 ± 4.79^c^	151.3 ± 10.54^a^	87.91 ± 9,74^bc^	110.50 ± 8.31^b^	102.00 ± 7.08^bc^	87.47 ± 6.62^bc^	89.06 ± 7.04^bc^
AST (U/L)	22.23 ± 1.25^c^	31.97 ± 0.85^a^	25.78 ± 0.96^bc^	28.11 ± 0.55^ab^	26.68 ± 1.03^bc^	25.20 ± 1.01^bc^	28.36 ± 1.06^ab^
ALT (U/L)	23.22 ± 0.79^c^	35.61 ± 1.32^a^	26.95 ± 1.18^bc^	29.49 ± 0.42^b^	28.22 ± 2.17^bc^	21.68 ± 1.18^c^	37.13 ± 2.13^a^
AP (U/L)	623.9 ± 22.89^d^	1229.0 ± 18.93^a^	746.5 ± 27.59^c^	1013 ± 30.87^b^	1047 ± 16.06^b^	1092 ± 62.06^b^	1002 ± 53.21^b^

Values are expressed as mean ± SEM, *n* = 6. Values in a line that do not share the same superscript letter are significantly different (*P* < 0.05) when one variable at a time was compared by one-way ANOVA followed by Newman-Keuls´ test

Glucose: F (DFn, DFd): 0.8397 (6, 45), P value: 0.5460; Triglycerides: F (DFn, DFd): 3.616 (6, 56), P value: 0.0042; Total cholesterol: F (DFn, DFd) 1.854 (6, 55), P: 0.0040; Uric acid: F (DFn, DFd): 1.470 (4, 29), P value: 0.0139; AST: F (DFn, DFd): 0.6245 (4, 25), P value: 0.0145; ALT: F (DFn, DFd): 1.289 (4, 29); P value: 0.0384; AP: F (DFn, DFd): 0.6291 (4, 26), P value: 0.0234

### Histological analysis, NAS score, lipid content and enzyme activities involved in lipid metabolism of liver tissue

**Fig. 3:**
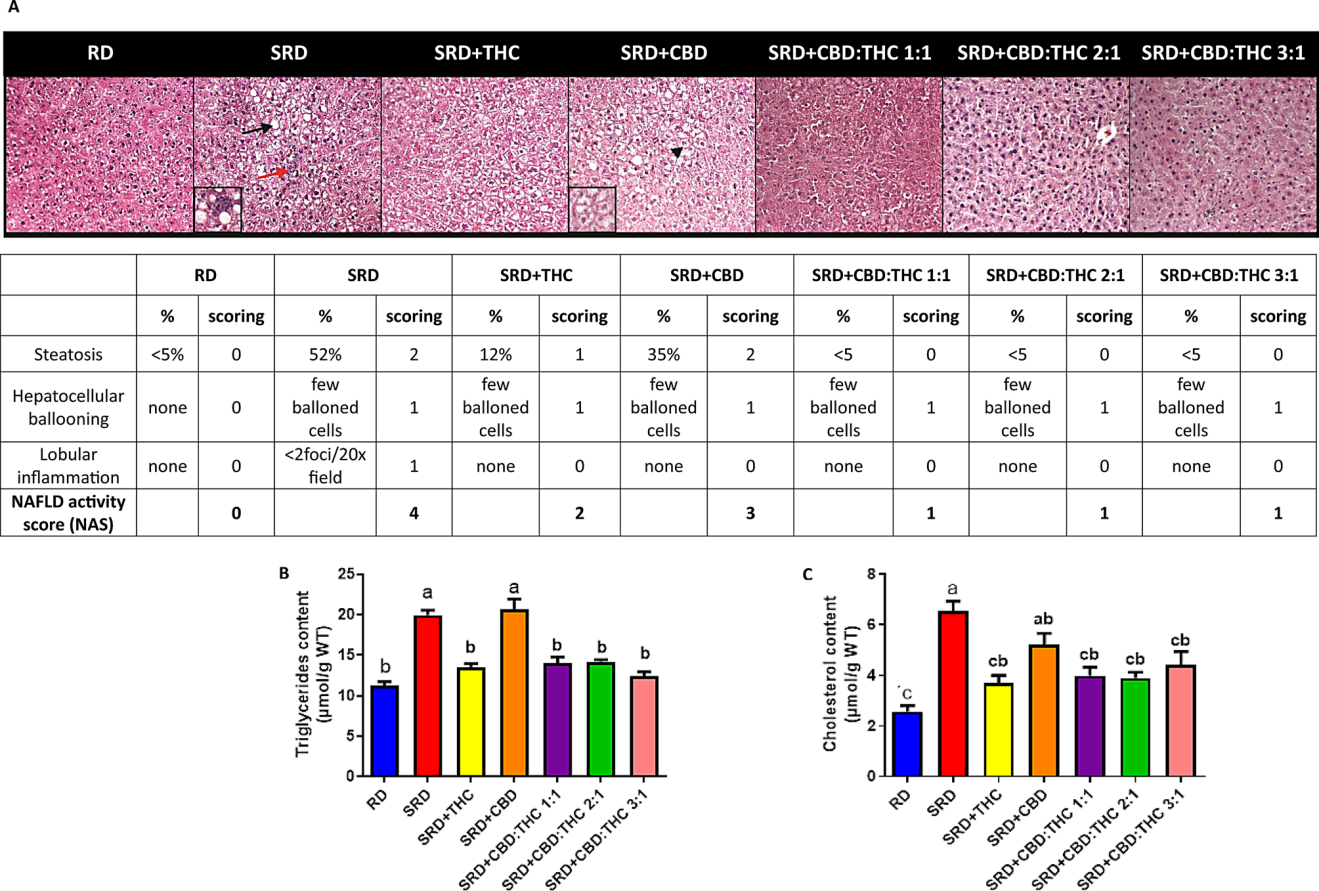
Histological analysis, NAS score and lipid content in liver tissue. **A**) Representative photomicrograph of histological abnormalities observed in liver sections. The accumulation of lipid droplets (black arrow), ballooning cells (black arrowhead) and inflammatory foci (red arrow) are observed in the liver section H&E-stained. 400× magnification. An inset is used to show, at high magnification, lipid droplets and inflammation foci in the SRD group, and hepatocellular ballooning in the SRD + CO₂ group. Table insert. Histologic scoring system for activity grade of nonalcoholic fatty liver disease (NAS) in liver sections. **B**) Triglycerides content. **C**) Cholesterol content. Values are expressed as mean ± SEM, *n* = 6. Bars that do not share the same letter are significantly different, (*P* < 0.05), when one variable at a time was compared by one-way ANOVA followed by Newman-Keuls´ test. Triglycerides content: F (DFn, DFd): 1.191 (4, 29), P value: 0.0356; Cholesterol content: F (DFn, DFd): 1.313 (6, 33), P value: 0.0279

Figure 3A shows an abnormal accumulation of lipid droplets within the cytoplasm of hepatocytes and infiltration of inflammatory cells in the histological sections and NAS score (Table insert in Fig. 3A) was higher in the SRD and SRD + CBD groups. These results were accompanied by a significant increase (*P* < 0.05) in triglycerides and cholesterol content in the liver tissue (Fig. 3B and C). SRD + THC, SRD + CBD:THC 1:1, SRD + CBD:THC 2:1 and SRD + CBD:THC 3:1 groups improved the abnormalities of the histological sections. In the SRD + THC, SRD + CBD:THC 1:1, SRD + CBD: THC2:1 and SRD + CBD:THC 3:1 groups, NAS decreased significantly (*P* < 0.05) (Table insert in Fig. 3A). The triglycerides and cholesterol content decreased significantly (*P* < 0.05), reaching reference values.

Acetyl CoA carboxylase (ACC) (4A), fatty acid synthase (FAS) (4B), malic enzyme (ME) (4C), and glucose-6-phosphate dehydrogenase (G-6-P DH) (2D) activities (key enzymes related to the novo fatty acids synthesis) were increased in the liver of SRD and SRD + CBD groups, compared with those of the RD-fed rats. The activities of ACC and ME decreased significantly (*P* < 0.05) in SRD + THC, SRD + CBD:THC 1:1, SRD + CBD:THC 2:1 and SRD + CBD:THC 3:1 groups and returned to values similar to those recorded in RD-fed rats. SRD + THC, SRD + CBD:THC 1:1, SRD + CBD:THC 2:1, SRD + CBD:THC 3:1 groups showed a significant reduction in FAS and G-6-P DH activities, although the values were still higher than in the RD group.

**Fig. 4:**
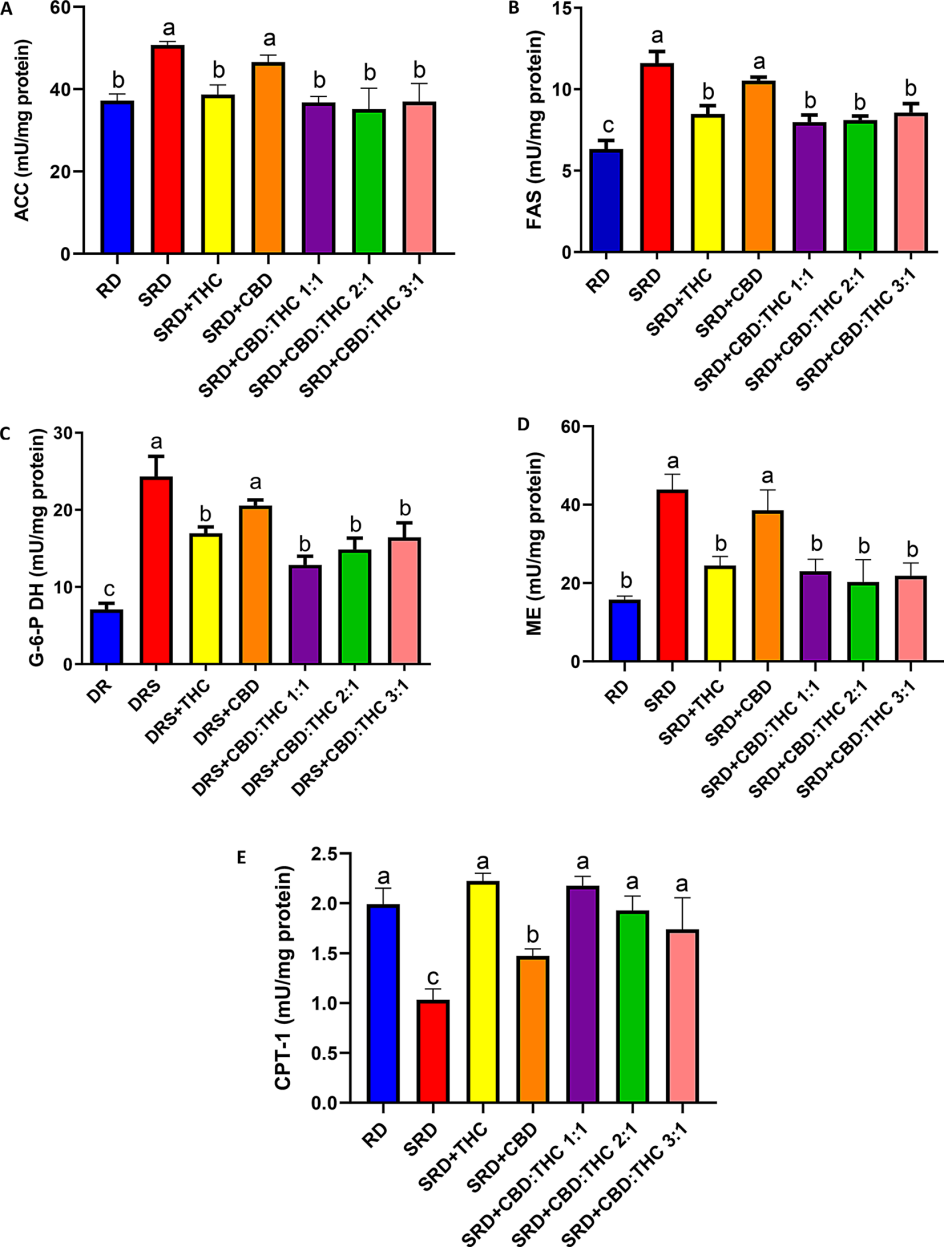
Enzymes involved in hepatic steatosis. **A**) Acetyl CoA carboxylase (ACC). **B**) Fatty acid synthase (FAS). **C**) malic enzyme (ME). **D**) glucose-6-phosphate dehydrogenase (G-6-PDH). **E**) carnitine palmitoyltransferase-1 (CPT-1). Values are expressed as mean ± SEM, *n* = 6. Bars that do not share the same letter are significantly different, (*P* < 0.05), when one variable at a time was compared by one-way ANOVA followed by Newman-Keuls´ test. ACC: F (DFn, DFd): 0.5965 (6, 30), P value: 0.0307; FAS: F (DFn, DFd): 0.3750 (4, 23), P value: 0.0241; ME: F (DFn, DFd): 1.308 (6, 32), P value: 0.0221; G-6-PDH: F (DFn, DFd):1.139 (6, 32), P value: 0.0325; CPT-1: F (DFn, DFd): 1.099 (6, 30), P value: 0.0386

On the other hand, Fig. 4E shows the hepatic activity of key enzyme related to mitochondrial fatty acid oxidation (CPT-1) in all dietary groups. THC-rich CO, CBD:THC 1:1 ratio CO, CBD:THC 2:1 ratio CO and CBD:THC ratio 3:1 CO administration prevented the decreased activity of CPT-1 observed in SRD-fed rats for 3 weeks, reaching values similar to those recorded in the RD. CBD-rich CO administration improved CPT-1 activity, although the values were still lower than in the RD group.

### Liver oxidative stress biomarkers

**Fig. 5:**
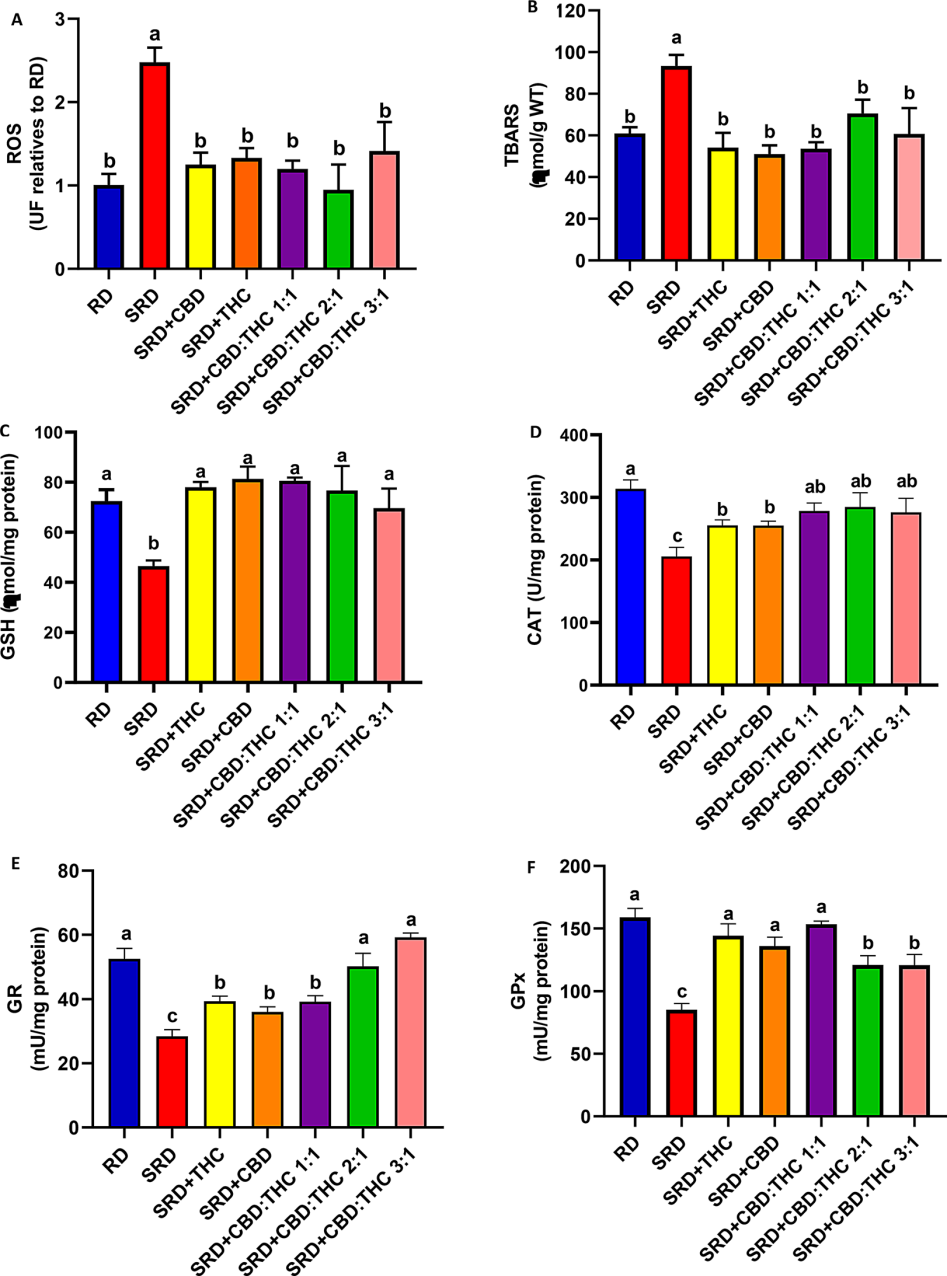
Liver oxidative stress biomarkers. **A**) reactive oxygen species (ROS). **B**) Thiobarbituric acid reactive substance (TBARS). **C**) glutathione (GSH). **D**) catalase (CAT). **E**) glutathione peroxidase (GPx) and **F**) glutathione reductase (GR). Values are expressed as mean ± SEM, *n* = 6. Bars that do not share the same letter are significantly different, (*P* < 0.05), when one variable at a time was compared by one-way ANOVA followed by Newman-Keuls´ test. ROS: F (DFn, DFd): 1.377 (6, 31), P value: 0.0254. TBARS: F (DFn, DFd):1.872 (6, 36), P value: 0.1127; GSH: F (DFn, DFd): 1.778 (6, 36), P value: 0.0131; CAT: F (DFn, DFd): 1.407 (6, 37), P value: 0.0237; GPx: F (DFn, DFd): 1.199 (6, 32), P value: 0.0332; GR: F (DFn, DFd): 1.633 (6, 32), P value: 0.0170

Figure 5A and B shows that liver ROS and TBARS were significantly increased (*P* < 0.05) in the SRD group compared to the RD group. When CBD-rich CO, THC-rich CO, CBD:THC ratio 1:1 CO, CBD:THC ratio 2:1 CO and CBD:THC ratio 3:1 CO were administered in the SRD, these parameters decreased significantly (*P* < 0.05) reaching similar values to those of the RD group. In addition, the decrease in the GSH content in the liver of the SRD group was increased (*P* < 0.05) in the SRD + CBD, SRD + THC, SRD + CBD:THC 1:1, SRD + CBD:THC 2:1 and SRD + CBD:THC 3:1 groups, reaching values similar to those of the RD group (Fig. 5C). Moreover, a significant decrease in CAT, GPx and GR activities was observed in the SRD group (*P* < 0.05). CBD-rich CO and THC-rich CO increased the CAT activity, although the values were still lower than in the RD group. CBD:THC ratio 1:1 CO, CBD:THC 2:1 ratio CO and CBD:THC ratio 3:1 CO increased the CAT activity, reaching values similar to those of the RD group (Fig. 5D). GR activity was increased (*P* < 0.05) in SRD + CBD, SRD + THC and SRD + CBD:THC 1:1 groups, although the values were still lower than in the RD group. In contrast, the SRD + CBD:THC 2:1 and SRD + CBD:THC 3:1 groups showed a further increase in GR activity, reaching levels similar to the RD group (Fig. 5E). In Fig. 5F was observed an increased GPx activity in SRD + CBD, SRD + THC and CBD:THC 1:1 groups, reaching values similar to those of the RD group. In SRD + CBD:THC 2:1, SRD + CBD:THC 3:1 groups, the GPx enzyme activity increased significantly, although the values were still lower than those in the RD group.

### Liver CB1 receptor

**Fig. 6:**
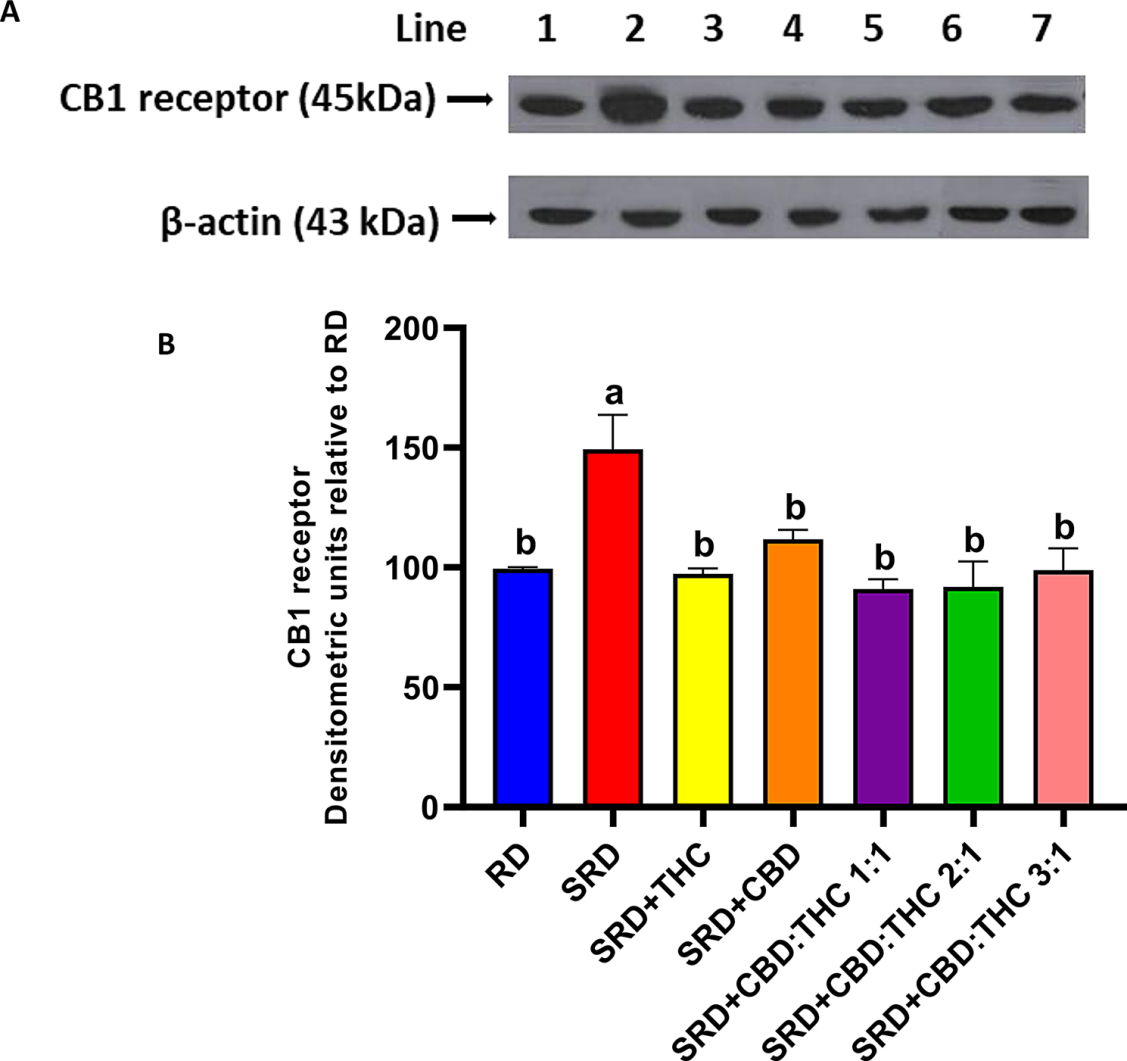
Liver CB1 receptor protein mass. Each gel contained an equal number of samples from rats fed a RD, SRD, SRD + THC, SRD + CBD, SRD + CBD:THC 1:1, SRD + CBD:THC 2:1 and SRD + CBD:THC 3:1. **A**. Representatives immunoblot of liver CB1 receptor. **B**. Densitometric immunoblot analysis of the CB1 receptor protein mass levels. Values are mean ± SEM (*n* = 6). Bars that do not share the same letter are significantly different, (*P* < 0.05) when one variable at a time was compared by one-way ANOVA followed by a Newman–Keuls test. CB1: F (DFn, DFd): 5.065 (6, 30), P value: 0.0011

Figure 6 shows the qualitative and quantitative results of the western blot, which indicate that CB1 receptor protein mass levels in the liver of rats fed with SRD were higher than those in the RD group (*P* < 0.05). Interestingly, when CBD-rich CO, THC-rich CO, CBD:THC 1:1 ratio CO, CBD:THC 2:1 ratio CO and CBD:THC 3:1 ratio CO were administered in the SRD, a significant decrease (*P* < 0.05) in the CB1 receptor protein mass levels was recorded, reaching values similar those to in the RD group (Fig. 6B).

## Discussion

The present study demonstrated that SRD-fed rats developed hypertension, dyslipidemia, liver damage, hepatic steatosis, lipid peroxidation, and oxidative stress. This was accompanied by upregulation of liver CB1 receptor expression. CBD-rich CO, CBD:THC 1:1 ratio CO; CBD:THC 2:1 ratio CO and CBD:THC 3:1 ratio CO showed antihypertensive properties. THC-rich CO, CBD:THC 1:1 ratio CO; CBD:THC 2:1 ratio CO showed the greatest beneficial effects against hepatic steatosis and liver damage. All COs exhibited antioxidant effects in liver tissue. This was associated with normal liver CB1 receptor expression (Table 5).

**Table 5:**
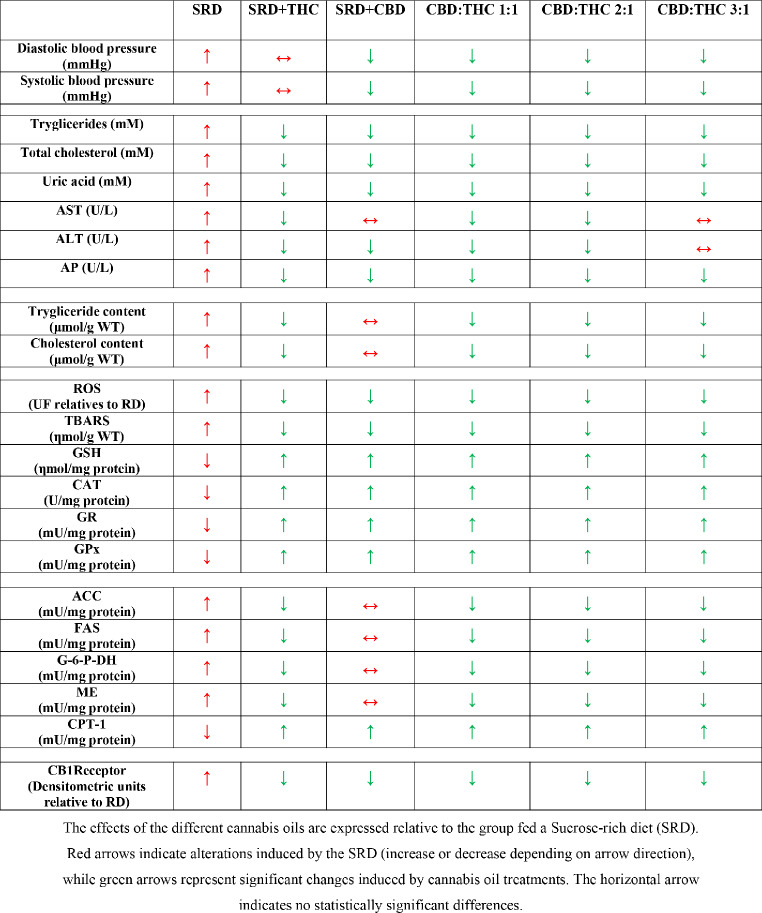
Summary of the main results

Hypertension is a hallmark of MS and is frequently associated with NAFLD, contributing to an elevated risk of cardiovascular complications. In this study, systolic and diastolic blood pressure levels were significantly increased in SRD-fed rats for 3 weeks, which mirrors the hypertensive phenotype often associated with NAFLD. Interestingly, CBD-rich CO and with varying CBD:THC ratios, such as 1:1, 2:1, and 3:1, demonstrated significant antihypertensive effects, restoring blood pressure values to near reference levels. This suggests that CBD may help alleviate the hypertensive component of MS, potentially due to its vasodilatory properties, as previously documented (Degrave et al. 2023b; Karimian Azari et al. 2020; Rajesh et al. 2010). In addition to cannabinoids, terpenes present in COs, such as myrcene, d-limonene, and linalool, may also contribute to these antihypertensive effects. These terpenes can improve vascular function through mechanisms such as nitric oxide release, vascular smooth muscle relaxation, and modulation of calcium channels, all of which are crucial in blood pressure regulation. The “entourage effect” between CBD and terpenes may further enhance these cardiovascular benefits by promoting vasodilation and modulating vascular tone (Silva et al. 2019; Alves Silva et al., 2016; Russo 2011). In contrast, THC-rich CO did not confer similar cardiovascular protection, highlighting a differential role of THC in blood pressure regulation. These findings align with previous research suggesting that CBD, either alone or in combination with certain terpenes, may have a more substantial impact than THC in lowering blood pressure and improving vascular function (Vallée 2023; Sultan et al. 2020; Jadoon et al. 2017; Russo 2011).

Dyslipidemia, a characteristic of MS and NAFLD, is strongly associated with impaired lipid metabolism and hepatic steatosis. In our study, SRD-fed rats exhibited significantly higher levels of triglycerides, cholesterol, liver damage markers (AST, ALT, and AP), elevated activity of enzymes involved in *de novo* lipogenesis and reduced CPT-1 activity, a marker of impaired fatty acid oxidation in NAFLD. Steatogenic agents such as ethanol, high-fat diet or sucrose rich diet can upregulate the activity of CB1 receptor via increasing synthesis of endocannabinoids, 2-arachidonyl glycerol (2-AG), and anandamide. CB1 receptor activation results in upregulation of lipogenic transcription factor and its target enzymes ACC and FAS and concomitantly downregulation of CPT-1. This leads to increase *de novo* fatty acid synthesis as well as decreased fatty acid oxidation, culminating into the development of fatty liver (Bazwinsky-Wutschke et al. 2019; Silvestri and Di Marzo 2013; Silvestri et al. 2011; Purohit et al. 2010; Osei-Hyiaman et al. 2005). Interestingly, while the CBD-rich and the CBD: THC 3:1 ratio COs did not improve lipid profiles, the THC-rich, CBD:THC 1:1 and CBD:THC 2:1 ratio COs significantly reduced triglycerides and cholesterol levels, improved liver histology, reduced NAS scores, and restored lipid balance, indicating their therapeutic potential in NAFLD. Notably, the THC-rich, CBD:THC 1:1, and CBD:THC 2:1 ratio COs reduced the activity of enzymes involved in de novo lipogenesis and enhanced hepatic fat oxidation. The modulation of lipid metabolism through cannabinoid signaling, particularly via CB1 receptor pathways, emerged as a key mechanism. In these line, Assa Glazer et al. (2020) demonstrated that the treatment with THC-rich or similar concentrations CBD:THC extract alleviated NAFLD development. The limited metabolic efficacy observed with the CBD-rich formulation may be related to the CBD dose used in this study. Indeed, the dose–response relationship of CBD remains controversial, particularly regarding its effects on lipid metabolism and liver function. While several studies have shown that CBD can exert beneficial effects on hepatic lipid accumulation and inflammation at moderate doses [3–20 mg/kg] (Flores-Cortez et al. 2025, Jiang et al. 2021; Silvestri et al. 2015), others have reported no significant effects [5–100 mg/kg] (Zandani et al. 2020, Kutanzi et al. 2020; Wang et al. 2017) or even potential hepatotoxicity at high doses [> 100 mg/kg] (Ewing et al. 2019; Watkins et al., 2021; Lo et al. 2023). However, it is important to note that none of these studies evaluated full-spectrum CO as used in the present study, which may involve an “entourage effect” from other bioactive components such as terpenes and cannabinoids. These findings highlight the need for further investigation into optimal cannabinoid ratios and dosing strategies, particularly for CBD, to balance efficacy and safety in the context of NAFLD. Moreover, terpenes have been reported to exert beneficial effects on lipid metabolism and to reduce hepatic steatosis in animal models of NAFLD. Several preclinical studies have shown that the administration of terpene-rich CO can lead to significant improvements in liver lipid accumulation and circulating lipid profiles. These effects are associated with reductions in hepatic fat deposition and modulation of key enzymes involved in the synthesis of triglycerides in animal models (Kim et al. 2024; Ghasemi-Gojani et al. 2023; Jing et al. 2013; Victor Antony Santiago et al. 2012).

Oxidative stress is a key contributor to the progression from simple hepatic steatosis to more severe forms of liver injury, such as NASH, where increased ROS production exacerbates inflammation, hepatocyte damage, and fibrosis (Arroyave-Ospina et al. 2021; Chen et al. 2020). In the present work, SRD group showed a significant increase in hepatic ROS and TBARS levels, accompanied by a marked depletion of GSH and reduced activities of key antioxidant enzymes, including CAT, GPx, and GR. These findings align with established NAFLD models, where high-sugar diets amplify oxidative stress by promoting lipid peroxidation and impairing the antioxidant defense system. Oxidative stress is a central driver in NAFLD progression, contributing to mitochondrial dysfunction, excessive ROS production, and sustained hepatocyte injury (García-Berumen et al. 2019; Jensen et al. 2018; Prasad and Dhar 2014). Conversely, the CO administration (SRD + CBD-rich CO, SRD + THC-rich CO, CBD:THC 1:1 CO, CBD:THC 2:1 CO and CBD:THC 3:1 CO) significantly reduced hepatic ROS and TBARS levels while enhancing GSH content and the activities of antioxidant enzymes (CAT, GPx, and GR). These results are consistent with previous studies highlighting the potent antioxidant properties of cannabinoids, particularly THC and CBD, in mitigating oxidative damage (Pereira et al. 2021; Rajesh et al. 2010; Hampson et al. 1998). In this line, Yang et al. (2014) demonstrated that CBD treatment effectively reversed lipid accumulation and liver damage, attenuated the increase in hepatic 4-Hydroxynonenal (4-HNE) levels induced by acute ethanol administration in mice. Similarly, Erukainure et al. (2021) showed that treatment with *Cannabis sativa* extracts significantly mitigated oxidative damage and enhanced both GSH levels and catalase activity in liver tissue exposed to FeSO_4_ in vitro. Furthermore, terpenes, a key component of CO, have demonstrated strong antioxidant and anti-inflammatory properties (Masyita et al. 2022; Downer 2020; Nuutinen et al. 2018). In this context, several preclinical studies have demonstrated that terpenes exert antioxidant effects in experimental models of liver damage and metabolic disturbances (Ahmad et al. 2018; Bacanlı et al., 2017; Baldissera et al. 2017; Basha and Sankaranarayanan 2016; Ciftci et al. 2011). Terpene administration has been associated with increased levels of endogenous antioxidant molecules such as GSH, and enhanced activity of key antioxidant enzymes including CAT, SOD, and GPx. These changes were accompanied by a reduction in markers of oxidative stress and lipid peroxidation. Overall, these findings support the role of terpenes in mitigating oxidative damage and preserving hepatic antioxidant defenses under conditions of metabolic or toxic challenge.

The CB1 receptor plays a crucial role in regulating lipid metabolism, and its activation has been shown to promote lipogenesis and impair fatty acid oxidation, leading to the accumulation of fat in the liver. Over the last few years, the ECS has attracted significant attention due to the connection between endocannabinoids, their lipid analogues, and various redox-dependent processes (Jorgačević et al. 2021; Lipina and Hundal 2016). Activation of the ECS via CB1 modulation likely underpins these metabolic effects, consistent with previous reports linking CB1 signaling to impaired energy balance, oxidative stress, and disrupted lipid metabolism (Silvestri and Di Marzo 2013; Rajesh et al. 2012). Furthermore, oxidative stress and the development of liver fibrosis are intricately related to CB1 receptor activation. By increasing the production of ROS and diminishing antioxidant defenses, CB1 receptor activation exacerbates oxidative stress, contributing to hepatocyte damage and the progression of fibrosis (Tan et al. 2020; Rivera et al. 2020; Bazwinsky-Wutschke et al. 2019; Mallat et al. 2013). Our results further support these findings, as we observed a significant overexpression of CB1 receptor protein mass levels in the liver of SRD-fed rats, consistent with previous studies linking high-fat/high-sugar diets to increased CB1 receptor expression. This overexpression was associated with heightened oxidative stress, lipid peroxidation, and markers of fibrosis, reinforcing the role of CB1 receptor in the progression of hepatic steatosis and related complications (Mallat et al. 2011; Osei-Hyiaman et al. 2005). These observations suggest that the pathological upregulation of CB1 receptor contributes to the metabolic and redox imbalances underlying NAFLD, highlighting its potential as a therapeutic target.

In the present study, the administration of COs (CBD-rich CO, THC-rich CO, CBD:THC 1:1 ratio CO, CBD:THC 2:1 ratio CO and CBD:THC 3:1 ratio CO) significantly reduced liver CB1 receptor protein mass levels. This reduction was accompanied by marked improvements in hepatic steatosis, oxidative stress, and fibrosis, except for lipid metabolism (lipogenesis and fatty acid oxidation) in the SRD + CBD group. The less pronounced effect observed in this group may be attributed to the specific properties of the CBD-rich formulation (SRD + CBD), which, while exhibiting potent antioxidant and anti-inflammatory effects, it may not influence the key pathways of lipid metabolism as effectively as other formulations. The decrease in CB1 receptor expression likely contributed to the observed restoration of lipid metabolism and redox balance, as previous studies have demonstrated that downregulation or inhibition of CB1 receptor can reverse steatosis, reduce oxidative damage, and mitigate fibrotic processes (Jorgačević et al. 2021; Gruden et al. 2016; Silvestri and Di Marzo 2013). Some authors suggest that the loss of CB1 receptor in mouse liver or the administration of CB1 receptor antagonists can reduce hepatic steatosis, as CB1 can be blocked by THC or CBD from cannabis (Chen and Kim 2024; Dibba et al. 2018). Although THC theoretically acts as an agonist of CB1 receptor, repetitive THC use may induce tolerance and downregulate CB1 receptor expression, potentially explaining the dose-dependent inverse relationship between marijuana use and NAFLD occurrence reported in other studies (Hirvonen et al. 2012; Le Strat and Le Foll 2011; Pertwee 2008). These mechanisms align with our findings, as the modulation of the endocannabinoid system, particularly the reduction in CB1 receptor density, likely contributed to the restoration of lipid metabolism, reduction of oxidative damage, and mitigation of fibrotic processes observed in our experimental model.

The role of terpenes in modulating the endocannabinoid system (ECS) has gained attention in recent years. Although the terpene content was broadly similar across all CO formulations, the presence of β-caryophyllene, humulene, guaiol, and bisabolol—all with reported anti-inflammatory and antioxidant properties—may have contributed to the observed effects through synergistic interactions with cannabinoids. This concept, known as the “entourage effect,” suggests that terpenes can enhance or modulate the actions of cannabinoids, improving therapeutic efficacy (André et al. 2024; Yao and Liu, 2023; LaVigne et al. 2021; Russo 2011). For instance, β-caryophyllene is a selective CB2 agonist with known hepatoprotective effects, which could complement the reduction in CB1 receptor expression observed in our study (Gertsch et al. 2008). However, due to the lack of significant differences in terpene composition among the oils, it is not possible to attribute specific outcomes to individual terpenes. Our findings instead support the hypothesis that a consistent profile of bioactive terpenes, acting in concert with THC and CBD, may contribute to the overall efficacy of cannabis-based treatments in NAFLD.

One of the main limitations of this study lies in the use of full-spectrum cannabis oils, which, although they have a well-characterized profile of cannabinoids and terpenes, do not allow us to discriminate the specific effect of each of their components on the ECS. Future studies should focus on the evaluation of isolated cannabinoids or terpene-rich essential oils to directly determine their impact on hypertension, lipid metabolism, oxidative stress, and CB1 receptor expression in an experimental model of NAFLD. It would also be beneficial to conduct further analysis of other related key components of the ECS, such as endocannabinoids, additional receptors or specific enzymes. These studies would be fundamental for a more comprehensive understanding of the mechanisms involved in the proposed experimental model.

## Conclusion

This study demonstrated that COs, particularly THC-rich formulations, and those with CBD:THC ratios of 1:1 and 2:1, effectively reduced dyslipidemia, hepatic steatosis, and liver damage in SRD-induced NAFLD. All COs exhibited significant antioxidant properties, which contributed to the attenuation of oxidative stress. Notably, oils containing CBD also displayed antihypertensive effects, likely due to their vasodilatory properties. The modulation of CB1 receptor is closely linked to the improvement in hepatic steatosis and oxidative stress. These results underscore the synergistic role of cannabinoids and terpenes in targeting key mechanisms involved in NAFLD pathophysiology.

These findings suggest that COs, especially those with balanced CBD: THC ratios (1:1 and 2:1) and with meaningful terpenes content, represent a promising therapeutic approach for managing NAFLD and preventing its progression to more severe liver diseases. Future studies are warranted to explore the precise mechanisms underlying these effects and confirm their clinical applicability.

## Electronic supplementary material

Below is the link to the electronic supplementary material.

Supplementary Material 1

## Data Availability

No datasets were generated or analysed during the current study.

## Abbreviations

ACC: Acetyl-CoA carboxylase
ALT: Alanine aminotransferase
AP: Alkaline phosphatase
AST: aspartate aminotransferase
CAT: Catalase
CB1: Type 1 cannabinoid
CBD: Cannabidiol
CPT-1: Carnitine palmitoyltransferase-1
CO: Cannabis oil
ECS: Endocannabinoid system
FAS: Fatty acid synthase
G-6-PDH: Glucose-6-phosphate dehydrogenase
GPx: Glutathione peroxidase
GR: Glutathione reductase
GSH: Glutathione
HFD: High fat diet
IHC: Immunohistochemistry
IOD: Integrated optical density
ME: Malic enzyme
MS: Metabolic síndrome
NAFLD: Non-alcoholic fatty liver disease
NAS: NAFLD activity score
NASH: Non-alcoholic steatohepatitis
RD: Reference diet
ROS: Reactive oxygen species
SRD: Sucrose-rich diet
SRD + THC: SRD plus oral administered THC-rich CO
SRD + CBD: SRD plus oral administered CBD-rich CO
SRD + CBD: THC 1:1:SRD plus oral administered CBD:THC 1:1
SRD + CBD: THC 2:1:SRD plus oral administered CBD:THC 2:1 CO
SRD + CBD: THC 3:1:SRD plus oral administered CBD:THC 3:1 ratio CO
TBARS: Thiobarbituric acid reactive substances
THC: Tetrahydrocannabinol
