# Supplementary material for: Effects of five cannabis oils with different CBD: THC ratios and terpenes on hypertension, dyslipidemia, hepatic steatosis, oxidative stress, and CB1 receptor in an experimental model
Source: J Cannabis Res. 2025 Jul 14;7:46. doi: 10.1186/s42238-025-00286-8 (PMC12261622; doi:10.1186/s42238-025-00286-8)
Supplement: Supplementary file 1 — Supplementary Material 1 [file 42238_2025_286_MOESM1_ESM.pdf]

Electrophoretic gels and blots

For your convenience, we have prepared the PDF of the information.

Each experiment includes sample lanes labeled with numbers (1 to 7).

The bands used for drawing are marked by red boxes.

line		sample	sample-blank	b-actin	b-actin-blank	Results	% control
	**blank**	116,62	30,03	117,74	15,61	1,9238	100,00
1	**RD**	146,65		133,35			
	**blank**	116,62	40,18	117,74	14,56	2,7596	143,45
2	**SRD 284**	156,8		132,3			
	**blank**	116,62	30,38	117,74	15,73	1,9313	100,39
3	**CO1 360**	147		133,47			
	**Bco**	116,62	30,27	117,74	16,12	1,8778	97,61
4	**CO2 366**	146,89		133,86			
	**Bco**	116,62	29,17	117,74	15,02	1,9421	100,95
5	**CO3 464**	145,79		132,76			
	**Bco**	116,62	29,83	117,74	15,35	1,9433	101,02
6	**CO4 457**	146,45		133,09			
	**Bco**	116,62	30,64	117,74	15,71	1,9504	101,38
7	**CO5 450**	147,26		133,45			

1 2 3 4 5 6 7

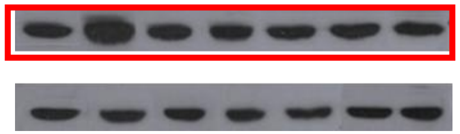

line		sample	sample-blank	b-actin	b-actin-blank	Results	% control
	**Bco**	116,67	22,11	93,57	37,65	0,5873	100,00
1	**DR 478**	138,78		131,22			
	**Bco**	116,67	18,6	93,57	37,01	0,5026	85,58
2	**CO2 367**	135,27		130,58			
	**Bco**	116,67	27,69	93,57	50,67	0,5465	93,06
3	**CO1 361**	144,36		144,24			
	**Bco**	116,67	25,89	93,57	45,38	0,5705	97,15
4	**CO3 465**	142,56		138,95			
	**Bco**	116,67	28,45	93,57	44,35	0,6415	109,24
5	**SRD 487**	145,12		137,92			
	**Bco**	116,67	22,54	93,57	40,43	0,5575	94,94
6	**CO4 458**	139,21		134			

1 2 3 4 5 6

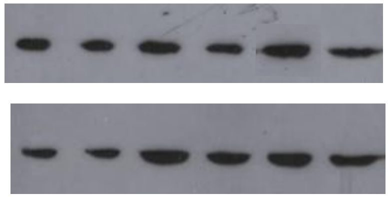

line		sample	sample-blank	b-actin	b-actin-blank	Results	% control
	**Bco**	126,37	33,77	110,76	16,03	2,1067	119,22
1	**CO2 368**	160,14		126,79			
	**Bco**	126,37	24,99	110,76	14,58	1,7140	97,00
2	**CO1 362**	151,36		125,34			
	**Bco**	126,37	32,09	110,76	18,16	1,7671	100,00
3	**DR 490**	158,46		128,92			
	**Bco**	126,37	35,93	113,25	19,4	1,8521	104,81
4	**CO3 466**	162,3		132,65			
	**Bco**	126,37	34,7	113,25	20,93	1,6579	93,82
5	**CO4 459**	161,07		134,18			
	**Bco**	126,37	36,57	113,25	24,18	1,5124	85,59
6	**CO5 452**	162,94		137,43			

1 2 3 4 5 6

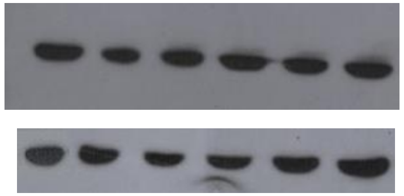

line		sample	sample-blank	b-actin	b-actin-blank	Results	% control
	**Bco**	121,23	37	119,71	21,29	1,7379	104,06
1	**CO2 369**	158,23		141			
	**Bco**	121,23	34,63	119,71	20,29	1,7068	102,20
2	**CO1 363**	155,86		140			
	**Bco**	121,23	26,37	119,71	15,79	1,6700	100,00
3	**DR 491**	147,6		135,5			
	**Bco**	121,23	20,19	119,71	14,39	1,4031	84,01
4	**CO3 467**	141,42		134,1			
	**Bco**	121,23	23,68	119,71	16,43	1,4413	86,30
5	**CO4 460**	144,91		136,14			
	**Bco**	121,23	26,49	119,71	17,38	1,5242	91,26
6	**CO5 453**	147,72		137,09			

1 2 3 4 5 6

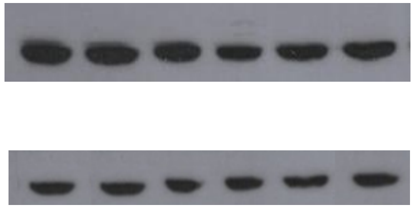

line		sample	sample-blank	b-actin	b-actin-blank	Results	% control
	**Bco**	124,4	37,18	112,02	37,69	0,9865	108,07
1	**CO2 370**	161,58		149,71			
	**Bco**	123	46,26	112,02	29,24	1,5821	173,32
2	**SRD 282**	169,26		141,26			
	**Bco**	130	35,39	109,45	38,77	0,9128	100,00
3	**DR 491**	165,39		148,22			
	**Bco**	123	41,56	111,02	43,13	0,9636	105,56
4	**CO3 468**	164,56		154,15			
	**Bco**	124,4	43,79	109,45	48,43	0,9042	99,05
5	**CO4 461**	168,19		157,88			
	**Bco**	123	42,67	109,45	45,65	0,9347	102,40
6	**CO5 454**	165,67		155,1			

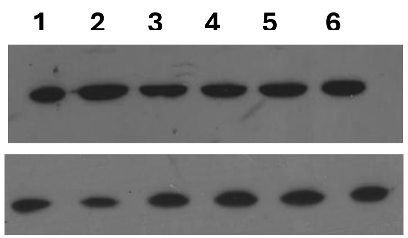

line		sample	sample-blank	b-actin	b-actin-blank	Results	% control
	**Bco**	130,69	30,86	111,3	41,65	0,7409	91,01
1	**CO5 451**	161,55		152,95			
	**Bco**	132,24	45,05	111,3	59,96	0,7513	92,29
2	**CO4 462**	177,29		171,26			
	**Bco**	131,69	25,9	111,3	38,05	0,6807	83,61
3	**CO3 469**	157,59		149,35			
	**Bco**	131,69	39,5	111,3	48,52	0,8141	100,00
4	**DR 480**	171,19		159,82			

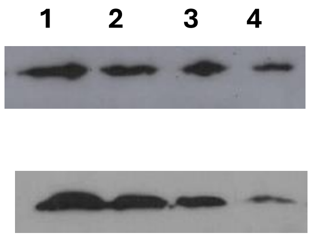

line		sample	sample-blank	b-actin	b-actin-blank	Results	% control
	**Bco**	123,52	20,67	119,98	20,29	1,0187	125,14
1	**SRD 486**	144,19		140,27			
	**Bco**	123,52	38,39	123,31	19,06	2,0142	108,55
2	**CO2 371**	161,91		142,37			
	**Bco**	123,52	34,54	123,31	18,23	1,8947	102,11
3	**CO1 364**	158,06		141,54			
	**Bco**	123,52	40,21	123,31	21,67	1,8556	100,00
4	**DR 490**	163,73		144,98			
	**Bco**	123,52	41,4	123,31	27,52	1,5044	81,07
5	**CO1 365**	164,92		150,83			
	**Bco**	123,52	47	123,31	31,29	1,5021	80,95
6	**CO5 455**	170,52		154,6			

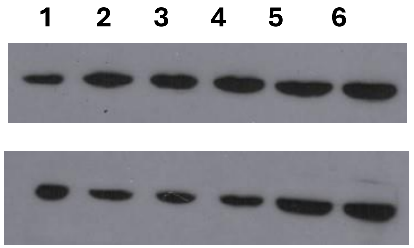

line		sample	sample-blank	b-actin	b-actin-blank	Results	% control
	**Bco**	134,67	10,84	110,42	26,38	0,4109	100,00
1	**DR 160**	145,51		136,8			
	**Bco**	134,67	14,39	110,42	23,76	0,6056	147,39
2	**SRD 483**	149,06		134,18			
	**Bco**	134,67	24,1	110,42	35,3	0,6827	166,15
3	**SRD 487**	158,77		145,72			
	**Bco**	134,67	21,72	110,42	33,66	0,6453	157,03
4	**SRD 488**	156,39		144,08			

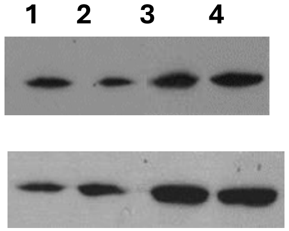

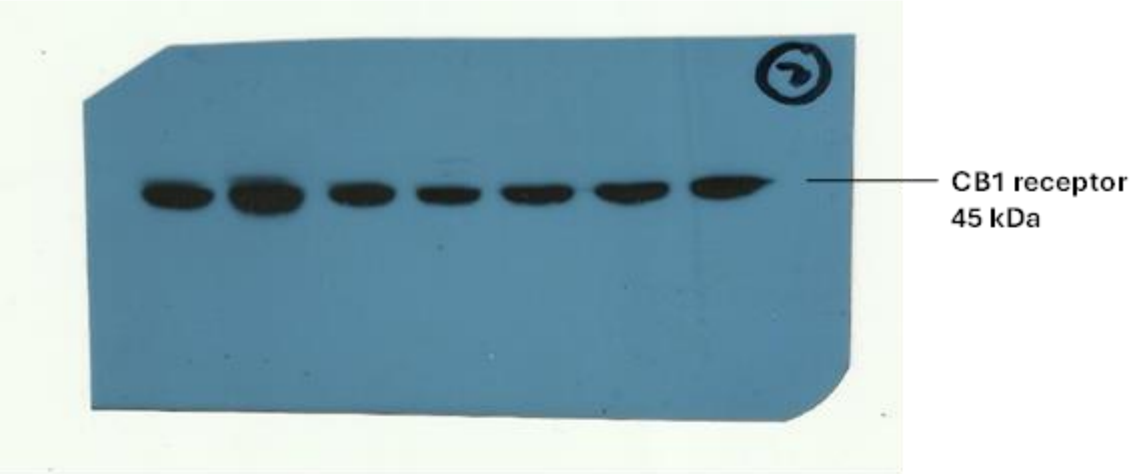
CB1 receptor
45 kDa

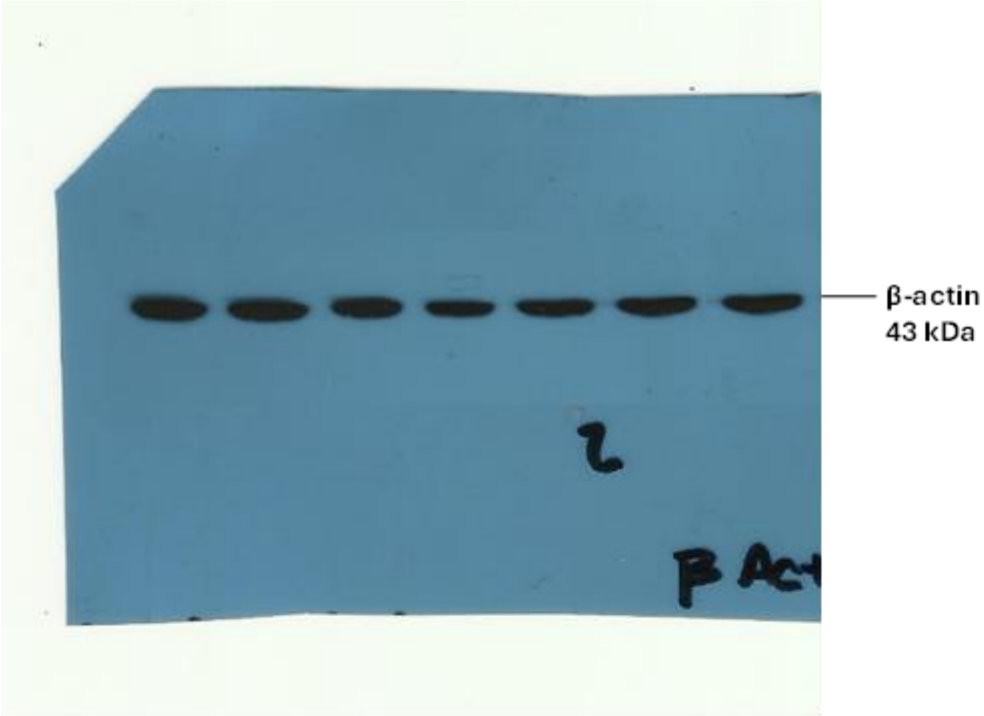
β-actin
43 kDa
